# A Convolutional Neural Network-Based Method for Discriminating Shadowed Targets in Frequency-Modulated Continuous-Wave Radar Systems

**DOI:** 10.3390/s22031048

**Published:** 2022-01-28

**Authors:** Ammar Mohanna, Christian Gianoglio, Ali Rizik, Maurizio Valle

**Affiliations:** Department of Electrical, Electronic and Telecommunication Engineering and Naval Architecture (DITEN), University of Genoa, Via Opera Pia 11, 16145 Genoa, Italy; christian.gianoglio@edu.unige.it (C.G.); ali.rizik@edu.unige.it (A.R.)

**Keywords:** radar, shadow effect, machine learning, CNN, transfer learning

## Abstract

The radar shadow effect prevents reliable target discrimination when a target lies in the shadow region of another target. In this paper, we address this issue in the case of Frequency-Modulated Continuous-Wave (FMCW) radars, which are low-cost and small-sized devices with an increasing number of applications. We propose a novel method based on Convolutional Neural Networks that take as input the spectrograms obtained after a Short-Time Fourier Transform (STFT) analysis of the radar-received signal. The method discerns whether a target is or is not in the shadow region of another target. The proposed method achieves test accuracy of 92% with a standard deviation of 2.86%.

## 1. Introduction

In recent years, the application of radars for target detection at short and medium ranges has become ubiquitous [1]. The use of short-range Ultra-Wide-Band (UWB) and Continuous-Wave (CW) radars is becoming an attractive solution for localization purposes. Some radar systems applications include through-wall and through-fire detection [2,3], the tracking of moving targets during security operations [4], the detection of trapped people after an avalanche or earthquake [5], and the detection, tracking, and classification of multiple targets passing through a security gate [6].

Up to now, the bi-static radars (with at least one transmitting antenna and at least one receiving antenna) have resolved the detection and localization of a single stationary target, yet the problem of multi-stationary target detection has been less addressed. The bi-static radars are able to accurately detect targets that are closer to the radar antennas, whereas the greater the distance of the targets from the radar, the lower the accuracy of the detection [7]. This is mainly attributed to two factors. Firstly, as the transmission distance increases, the energy of the electromagnetic wave is attenuated; hence, the energy of electromagnetic waves reaching farther targets is inevitably smaller than that reaching the closest target. Secondly, some targets, named recessive targets, can lie in the shadowed region of a dominant target (i.e., the closest to the radar). Thus, because the highest energy of the electromagnetic waves is reflected from the dominant target to the radar, the electromagnetic illumination of the recessive targets could decrease to the point where they are not detected [8]. Therefore, radar systems suffer from what is called the shadowing effect. This effect occurs when two targets stand in front of the antenna, one in the shadowing region of the other. The radar is usually not reliably capable of detecting the target that is standing in the shadow region [7]. This problem is common for most radar technologies, particularly, Ultra-Wide-Band (UWB) radar [2] and Frequency-Modulated Continuous-Wave (FMCW) radar [9]. Unlike pulse and Ultra-Wide-Band (UWB) radars, FMCW systems require lower sampling rates and lower peak-to-average power ratios to detect the distance and speed of multiple moving targets [10,11]. Accordingly, the FMCW radar is a good solution for detection and localization purposes but performs poorly whenever the shadow effect occurs. The shadow effect is more relevant in low-cost radars. This is due to their lower resolution compared to the high-end radars (higher range and velocity resolution) [12].

In the literature, the shadow effect has been targeted only by a few papers [2,7,8,13,14,15,16,17,18,19]. The authors proposed non-scalable solutions, thus requiring expert intervention for applying their methods in different environments. In this paper, we propose a novel solution for solving the issue of target identification in the shadow region and we adopt Deep Learning (DL) techniques. This method is quantitatively analyzed and results are presented in Section 6. It benefits from the promising achievements presented in the literature of applying AI techniques on post-processed radar data. These techniques can help to dynamically learn suitable filters. This proposed solution is also scalable and does not need expert intervention.

In general, DL methods have proven to be very efficient in real-world image classification [20]. Moreover, DL techniques that use radar input are adopted for a wide range of applications, such as target classification [21], object tracking [22], and gesture recognition applications [23]. Among DL techniques, Convolutional Neural Networks (CNNs) are particularly suited for addressing image processing problems [24,25]. Our proposed method uses a lightweight CNN model based on ImageNet (i.e., convolutional filters have been pre-learned based on ImageNet data [26]) to target the discrimination of shadowed targets, fine-tuning only the weights of the last layer (i.e., dense layer). The convolutional layers perform the feature extraction without any prior knowledge of the user. To validate the proposed solution, we address a two-class classification problem: one target vs. two targets. In the latter, one target is in the shadowing region of the other. Four models have been tested using the collected dataset. The best model in terms of accuracy is the MobileNet_V3 Large version; it achieves a generalization performance on the test set of 92.2%. The results encourage us to extend the adoption of CNNs in applications such as identifying and tracking more shadowed targets.

The rest of this paper is organized as follows. In Section 2, the state of the art of the targeted research domain is extensively presented. In the following Section 3, the problem statement is explained. Section 4 presents and discusses the methodology adopted to identify and solve the shadowing effect. The experimental setup is considered in Section 5, explaining the data acquisition process, time frequency analysis, and training process. The experimental results and discussion are presented in Section 6. Finally, the conclusions and some proposals for future work are provided in Section 7.

## 2. State of the Art

In [13], the shadow effect and its removal using PCL radar is investigated. A study on PCL radar performance under the shadowing effect is presented, when a distant, weak target echo is shadowed by strong echoes. In [7,8], the authors outlined the origin of the shadow effect as the impact of the mutual shadowing of targets in a multiple-person tracking scenario. This explanation is confirmed by the experimental measurements. Other researchers investigated the shadowing effect for the purposes of person detection and tracking with UWB radars [14]. The results confirm the existence of additional attenuation within the shadow zones. In [15], a technique based on wavelet entropy is proposed because of the significant difference in frequency ratio components between the echo signal of the tested target and that of the masked target generated by dynamic clutter. Wavelet entropy can accurately detect multiple human targets in the presence of dynamic clutter, even if the distant human targets are in the shadow area of the closer target, as compared to the reference techniques of adaptive line enhancement and energy accumulation. In [2], a significant difference in frequency was detected between the echo signal of the human target and that of noise in the shadowing region. The authors concluded that the target detection using the power spectrum is not effective. Therefore, an auto-correlation algorithm is applied to the pre-processed signals in order to compute the wavelet entropy. Results show that the proposed approach is capable of detecting a shadowed target. Other applications have been addressed in the literature [16,17,18,19]. In general, none of the previous works have presented a scalable solution for solving the shadowing effect. In fact, these solutions require expert intervention for applying them in different environments.

Several works involving the use of FMCW radar have been reported in the literature. In [23], the authors introduced a novel system for dynamic continuous hand gesture recognition based on a Frequency-Modulated Continuous-Wave radar sensor. They employed a recurrent 3D CNN to perform the classification of dynamic hand gestures and achieved a recognition rate of 96%. In [27], the authors proposed a prototype of an FMCW radar system for the classification of multiple targets passing through a road gate. The classification covered four classes: pedestrians, motorcycles, cars, and trucks. It achieved accuracy of 88.4%. Many other applications have been tackled in the literature [9,28,29,30,31,32,33,34,35]. Most of the systems presented in the aforementioned works suffer from the shadow effect. However, none of them have proposed a solution.

Deep learning classification techniques for radar target classification have also been adopted in the literature. The practical classification of a moving target system, based on automotive radar and deep neural networks, is presented in [36]. The study presents results for the classification of different classes of targets using automotive radar data and different neural networks. In addition, a human–robot classification system based on 25 GHz FMCW radar using micro-Doppler features was introduced in [37]. The raw Range-Doppler images were directly fed into a CNN, resulting in performance with 99% accuracy for distinguishing humans from robots. Many other applications that use neural networks for radar problems have been tackled in the literature [38,39,40,41,42].

## 3. Problem Statement

### 3.1. FMCW Radar Device

The multi-chirp FMCW algorithm is considered the standard for detecting and measuring the range and speed of multiple targets [43]. The concept of multi-chirp is to send a frame containing a number of chirps (Nc) in saw-tooth modulation and in a short period, with the chirp time (Tc) being very small (in μs), where Tf is the time of the data frame (Tf is in ms). In the current scenario, the “Position2Go” [44] cheap radar is used. It is an FMCW radar board developed by Infineon technologies. This development kit allows the user to implement and test several sensing applications at the 24 GHz ISM band, such as tracking and collision avoidance. This is possible by using fast chirp FMCW and two receiving antennas to obtain the angle, distance, speed, and direction of motion. The radar is equipped with a pair of arrays of microstrip patch antennas (one for transmitting and two for receiving) characterized by a 12 dBi gain and 19 × 76 degree beam-widths, defining the Field of View (FoV). The kit consists of the BGT24MTR12 transceiver MMIC and a XMC4700 32-bit ARM^®^ Cortex^®^-M4 for signal processing and communication via USB. The radar is connected via USB to a PC that is running MATLAB. A MATLAB script sends the order to the radar to initiate the data acquisition procedure through the USB port. Table 1 shows the radar sensor parameters.

### 3.2. Shadow Effect

**Figure 1:**
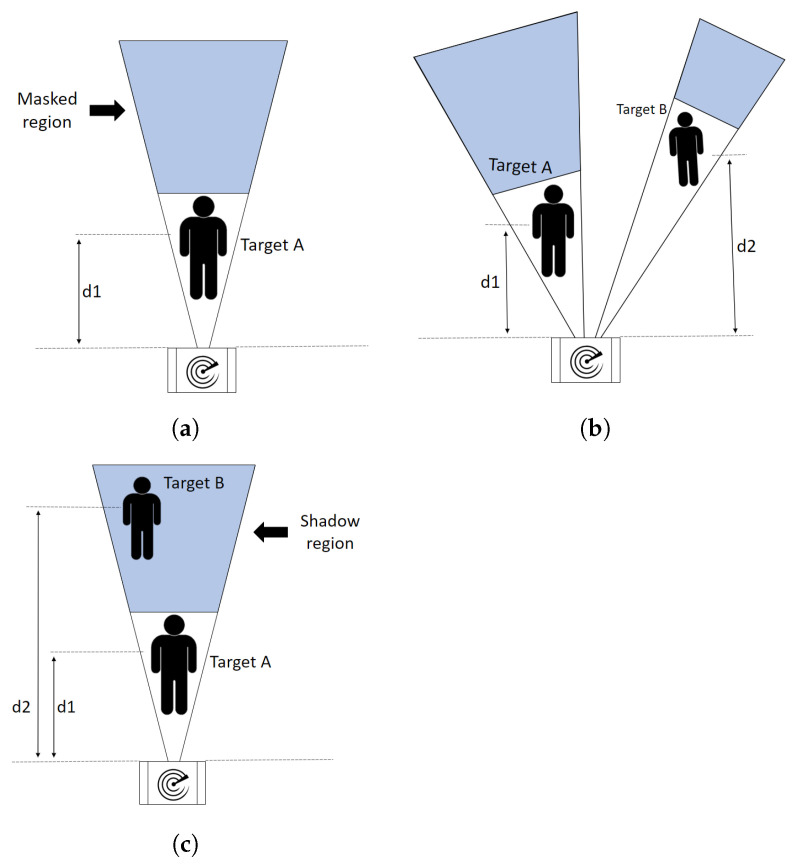
Illustration of the data collection setup. (**a**) One target in range of the radar. (**b**) Two targets in range of radar, both visible to the radar. (**c**) Two targets in range of radar, only one visible to the radar.

Figure 1 shows different cases of target detection using a radar. In particular, Figure 1a illustrates the case of a single target standing in the range of the radar, Figure 1b depicts two targets both detectable by the radar, while Figure 1c represents the shadowing effect where target B is masked by target A and thus target B is not visible to the radar.

**Figure 2:**
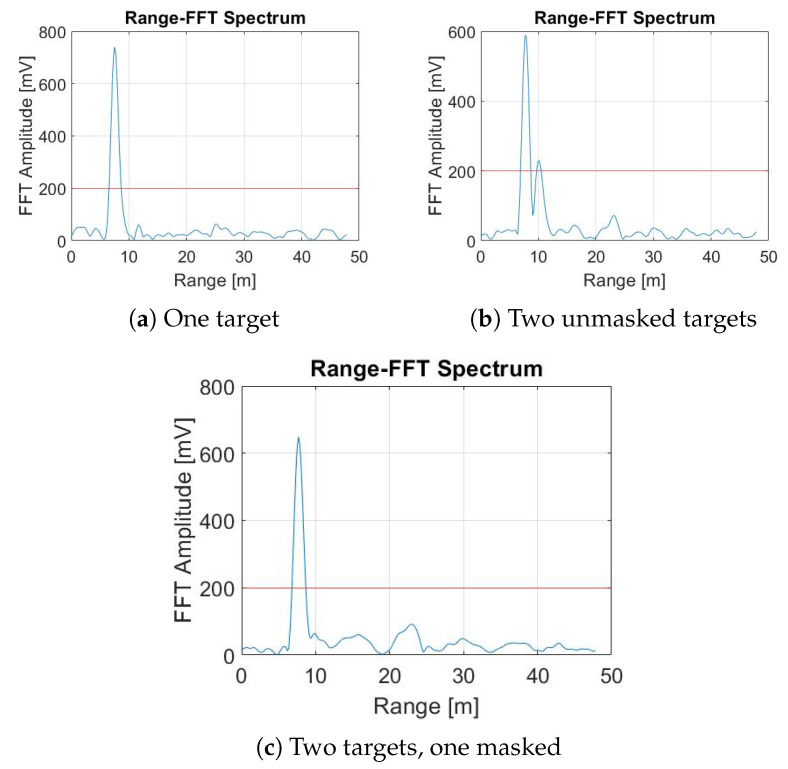
Range-FFT power spectrum. The horizontal red line is the target detection threshold. Radar is positioned 1.5 m from the floor. (**a**) Only target A (d1=7 m), (**b**) both targets A (d1=7 m) and B (d2=10 m) without shadowing effect, (**c**) target B (d2=10 m) shadowed by target A (d1=7 m).

The shadowing effect creates a region behind the target (Target A in Figure 1c) where the electromagnetic waves emitted by the radar transmitting antenna or reflected by another object are not able to propagate. In fact, computing the power spectrum on the multi-chirp data acquired by the FMCW radar, it is possible to detect the masked target, but the detection is accompanied by a lot of variability in the measurements. The reason for such variability is that a few radar waves penetrate and slip through the shadowing target to the masked one, reflecting to the radar with a very low intensity. These waves in particular cause huge variability that can affect the detection parameters of both targets in the Field of View (FoV) of the radar. Figure 2 shows three examples of the range representation obtained after the fast-time FFT (range-FFT) on multi-chirp signals [43], positioning the radar 1.5 m from the floor. Each target on the spectrum is represented by a peak. A fixed target detection threshold (red horizontal line) is used to determine the valid target identifications, i.e., each target passing in the FoV of the radar with a peak higher than the fixed threshold is considered a valid detection by the radar. The threshold is a user-defined parameter that affects radar performance directly by causing a trade-off between detection accuracy and false alarm probability. If it is chosen to be too high, the algorithm will fail to identify some targets. If it is too low, the algorithm will detect many artifacts as targets. Figure 2a shows only one peak at a distance of 7 m; this situation is illustrated in Figure 1a, where only one target is in front of the radar (d1=7 m). In Figure 2b, two peaks appear at distances of 7 m and 10 m, respectively; this spectrum is the result of a trial where two people were standing in different positions (i.e., d1=7 m and d2=10 m) with no shadow effect on each other, as illustrated in Figure 1b. The magnitude of the peak at a 10 m is smaller than that at 7 m because of the attenuation of the electromagnetic wave of the radar as the distance increases. In Figure 2c, the maximum peak appears at a distance of 7 m, which corresponds to the location of target A (d1=7 m). However, target B (d2=10 m) cannot be detected, since he stands in the shadowing region. An example is illustrated in Figure 1c.

Therefore, the traditional spectrum method is not reliable for detecting multiple targets where the shadow effect occurs. The shadowed targets are hardly detected. This fact is dependent on the chosen power threshold, Radar Cross-Section (RCS) of the shadowing target [45], and the environmental clutter.

In the case when target B is not fully shadowed by target A, target B is expected to be detected with a weak signal, based on how much it is shadowed by target A. However, this detection is also relative to the chosen detection threshold. The radar is capable of discontinuously detecting target B when it is not completely aligned with target A [7]. However, the detection of target B is not reliable, and the partial shadow effect was excluded from our testing campaign because it represents a simplified version of the full shadow effect illustrated in Figure 1c.

## 4. Methodology

To address the shadow effect, a novel approach is proposed. The idea is that a small portion of the waves slip through or around the shadowing target (target A in Figure 1c) towards the shadowed target (target B in Figure 1c). The masked target is receiving and reflecting these slithered electromagnetic waves, thus causing a slight but noticeable variation in the waves received by the radar. These reflections are used to identify whether there is a masked target or not. This goal could be achieved using time frequency analysis to construct images (i.e., spectrograms) that feed CNNs, addressing a two-class image classification problem (one target vs. two targets).

### 4.1. Time Frequency Analysis

Spectrograms are a popular signal processing tool used to reveal the instantaneous spectral contents of the time-domain signal. They also show the variations in the spectral content over time. A spectrogram is obtained by applying the squared magnitude of the STFT computed over a discrete input signal. The STFT can be formalized as:

(1) $$STFT\left\{x\left[n\right]\right\}=X\left(m,k\right)=\sum_{n=-\infty }^{\infty }x\left[n\right]w\left[n-m\right]e^{-j2\pi kn/N}$$

where x[n] is the discrete signal, w[n] is the discrete window function, which is non-zero in [0⋯N] and zero elsewhere, *N* is the number of samples in the window, and *k* is the discrete frequency. The window’s location is indicated by the index *m*. The spectrogram can be generated by continuously computing the STFT with increasing *m* by a step size Δm. The step size Δm can be used to achieve an overlap between two consecutive analysis windows, resulting in a smoother time dimension output. Eventually, to use the computationally quicker Fast Fourier Transform (FFT), a power of 2 must be selected for *N*, or *N* can be zero padded to a power of 2. As a rule of thumb, a large window size indicates a high resolution in the time domain and low resolution in the frequency and vice versa.

### 4.2. Deep Neural Network Models

To address the shadowing effect problem in its most simplified form, only two classes were considered in this study (one target and two targets). To address the two-class classification problem, we employed CNNs trained over the spectrogram images. The CNNs proved to be very efficient in image classification. In particular, MobileNet models are suitable for deployment on embedded systems since they achieve similar accuracies in the object classification problem, while requiring less parameters than ResNets and VGGs. The peculiarity of the MobileNet models is the adoption of the depth-wise separable convolution [46], i.e., the standard convolution operator is replaced by two separate layers: the first layer involves one convolutional filter per input channel, while the second is a point-wise convolution. For an input of size H×W×D, and a 2D convolutional layer presenting $N_{k}$ kernels of size K×K, the computational cost $C_{SC}$ of the standard convolution is:

(2) $$C_{SC}=H\times W\times D\times N_{k}\times K\times K$$

while, using the depth-wise separable convolution, the cost $C_{DSC}$ is:

(3) $$C_{DSC}=H\times W\times D\times \left(K^{2}+N_{k}\right)$$

which is significantly smaller than (2).

Table 2 shows a comparison of some state-of-the-art MobileNet models (i.e., V2 and Small V3) with ResNet50 and VGG19 networks [47], all trained on the Imagenet dataset. The first column reports the models, the second represents the number of parameters, the third shows the generalization accuracy on the Imagenet dataset, the fourth displays the model sizes in megabytes, while the last column presents the average inference time of the models running on GPU Tesla A100. The table demonstrates that the MobileNet models can achieve similar generalization performance, employing few parameters with respect to more complex models.

Following the results of Table 2, four different MobileNet-based architectures were compared. The four models were pre-trained on the Imagenet dataset; thus, the weights and biases were statically loaded, before eventually fine-tuning the last trainable dense layer using the collected dataset. Hence, the convolutional layers of the MobileNet models provided the filters, learned on the Imagenet dataset, to process the input image. Eventually, the features extracted by the convolutional layers were fed to the dense layer, which classified the data among the two possible classes (one target vs. two targets). The data collection procedure is presented in Section 5.1.

## 5. Experimental Setup

**Figure 3:**
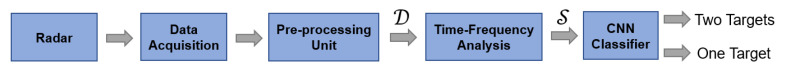
Block diagram of the proposed system.

Four persons were involved in a series of experiments with the aim of collecting data to validate the proposed solution. In the following section, the data retrieval process is described. In addition, the spectrogram hyperparameter selection is explained. Finally, the CNN training phase is described. A block diagram of the proposed system is illustrated in Figure 3.

### 5.1. Data Acquisition

In order to overcome the possible problem of the multi-path effect, a clutter removal technique proposed in [27] was used to remove the environmental clutter (i.e., the potential ghost effect) from the source.

**Figure 4:**
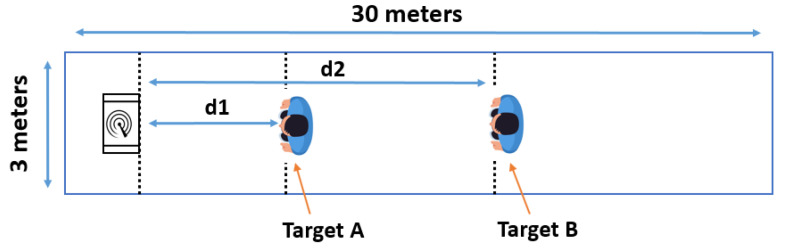
Illustration of the corridor data collection environment.

Two sets of experiments were carried out for this study. Measurements took place in a thirty-meter-long and three-meter-wide corridor. The corridor environment was chosen because it maximized the clutter, thus making it harder for the radar to detect the shadowed target. The goal of the experiments was to detect all human targets standing in range of the radar. The radar was placed one and a half meters from the ground. Figure 4 schematizes the experimental environment.

**Figure 5:**
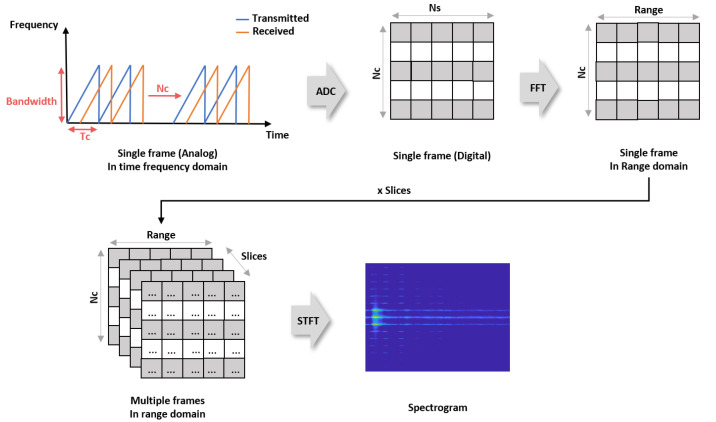
Data processing pipeline.

As illustrated in Figure 3, data are collected and pre-processed to reach a spectrogram format (i.e., image). These spectrograms are then fed to the CNN classifier. Figure 5 shows the data processing pipeline from the raw radar outcome towards the spectrogram format. The data corresponding to chirps are stored as the rows of a matrix of dimension Nc×Ns (i.e., Nc is the number of chirps and Ns is the number of samples of each chirp). To convert the data type, an Analog to Digital Converter (ADC) was used. Range FFT is then applied on each row, which results in a range representation. Multiple slices (Slices=50 in this study) of this matrix are then collected to form a tensor (Nc×Range×Slices). The slices are collected consecutively: as soon as the *n*-th slice is collected, the radar immediately starts to collect the slice *n*-th + 1. Finally, STFT is applied on this 3D tensor to obtain the spectrogram.

For the first experiment, illustrated in Figure 1a, a single target (target A) stood in range of the radar. The target was standing in different positions gradually through all reference positions d1 along an eleven-meter range. Four different human targets were involved in this experiment to increase the data diversity. One hundred and fifty measurements were collected for each target. Therefore, for the first experiment, six hundred measurements were collected. This dataset is called the One Target (OT) class.

For the second experiment, illustrated in Figure 1c, two targets, A and B, stood in range of the radar. Target A, who was closer to the radar antennas, was standing gradually through all reference positions d1 along the eleven-meter range in front of the antennas, and target B, who was farther away from the antennas, stood in the shadowing region of target A (setup is illustrated in Figure 1c), two meters behind him. Three persons were involved in this experiment, exchanging their mutual positions. Six hundred measurements were collected during this experiment; this dataset is called the Two Targets (TT) class. To sum up, the complete collected dataset consists of one thousand two hundred samples, divided in half among the two classes. The extracted dataset is formalized in:

(4) $$D=\{{\left(X,y\right)}_{i};\;X_{i}\in R^{Nc\;\times \;Ns\;\times \;Slices},\;y_{i}\in \left\{OT,TT\right\};i=1,\ldots ,Z=1200\}$$

where Nc=32, Ns=128, and Slices=50.

Table 3 summarizes the data collection setup. The first column represents the class (One Target vs. Two Targets). The second and the third columns present the distances from each target to the radar (i.e., target A and target B, respectively). The last column displays the number of measurements acquired for each combination of the targets involved in the experiments. In the case of the One Target class, 4 persons were involved (i.e., four combinations for each d1 distance); thus, there were 30 acquisitions per combination. For the Two Targets class, measurements were obtained on 3 persons exchanging their mutual position (i.e., 6 possible combinations for each pair [d1,d2]), hence leading to 20 acquisitions per combination.

### 5.2. Spectrogram

**Figure 6:**
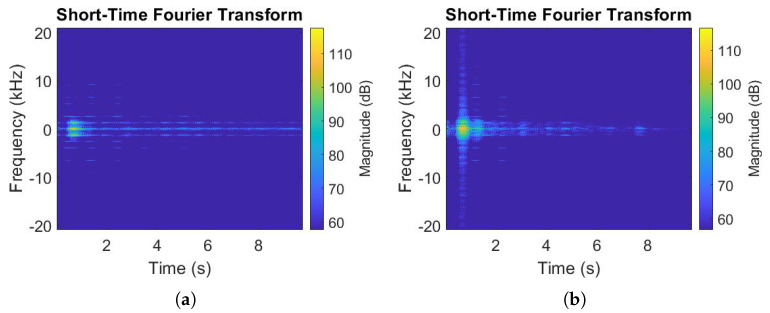
Spectrogram examples. (**a**) One target. (**b**) Two targets.

According to Section 4.1, a time-frequency analysis was carried out on D to extract spectrograms in order to feed CNNs. To obtain a continuous spectrogram, a large window size was chosen with N = 2048, with a 50% overlap (Δm = 1024). Two samples of the obtained results are illustrated in Figure 6.

The spectrograms of the 1200 collected samples were generated. The original dimensions of each spectrogram were (875, 656, 3); each was down-sampled and zero padded to fit the input size of our CNN, with the dimensions (224, 224, 3). The dataset containing spectrograms can be formalized as:

(5) $$S=\{{\left(\hat{X},y\right)}_{i};{\hat{X}}_{i}\in N^{224\times 224\times 3},\;y_{i}\in \left\{OT,TT\right\},i=1,\cdots ,Z=1200\}.$$

Figure 6a shows an example of the generated spectrogram for the One Target class as illustrated in Figure 1a. In this example, the target was standing five meters away from the radar. Figure 6b shows an example for the Two Targets class as illustrated in Figure 1c. In this example, target A was five meters away from the radar while target B was two meters behind target A, so seven meters away from the radar. If inspected carefully, a difference is visible in Figure 6a,b; this difference represents the passive electromagnetic waves reflected from target B and received by the radar antenna.

### 5.3. Training

The authors adopted the four most common implementations of the MobileNet architectures. The number of neurons in the trainable dense layer was set to 128 for each network, using the ReLU as a non-linear activation function. Moreover, the Stratified K-Fold technique was adopted to ensure fair results. Stratified sampling consists of splitting the data of the original labeled dataset (i.e., the population) into subsets, having the same proportion of data as in the population. The subgroups are called ‘strata’. Thus, adopting the stratified method in cross-validation guarantees that the training and test sets contain the same proportion of labeled dataset in each fold, leading to a close approximation of the generalization error on the test set. In each of the ‘K’ iterations of the K-fold cross-validation technique, where the data have been split into ‘K’ groups, one portion is used as the test set, while the remaining portions are used for training. In the current situation, ‘K’ was chosen to be equal to five. Therefore, five folds were generated, and results will be presented in the next section as the average of the five results from each of the folds. In this way, 80% of the data have been used for training and 20% for testing in each iteration run. Actually, the test data were split into two parts (validation and test sets) having the same number of samples. An early stopping criterion was implemented during training over the validation set, fixing the patience parameter to 10. All the results have been averaged over the 5 folds. The Adam optimizer function was used with a learning rate of 1/2000. Regarding the loss function, categorical cross entropy was used. Models were trained for one hundred epochs for each split.

## 6. Experimental Results and Discussion

The results achieved using the proposed approach are presented in Table 4. The first column provides the four adopted model architectures, the second shows the number of parameters, the third column reports the average accuracy computed on the test set of the five folds and the standard deviation for each model, the fourth column depicts the average inference time of the model running on a RTX-2080Ti GPU, while the last column presents the saved model sizes.

The table shows that all the MobileNet_V3-based networks generally perform better than MobileNet_V2. This could be explained by the introduction of the hard-swish activation function and the implementation of a squeeze-and-excitation module [46]. Among the three MobileNet_V3 versions (Large, Small, and Small Minimalistic), the testing accuracy results are directly proportional to the number of parameters used in the architecture: the higher the number of parameters, the higher testing accuracy is achieved.

A compromise should be taken when choosing the model. This compromise would be highly dependent on the application scenario. If the application scenario is critical and the accuracy is the main interest, Large MobileNet_V3 would be chosen. If the goal is to deploy on the edge, then memory and inference time would be the main goal, and Small Minimalistic MobileNet_V3 would be chosen. Usually, the main interest in using a low-cost radar is the possibility of edge deployment, and the main constraint of edge deployment is the number of parameters, i.e., the model size. Therefore, the Small Minimalistic MobileNet_V3 best suits the proposed use-case.

Under the proposed circumstances, where either one or two targets are in the detection range of the radar, the model choice (i.e., number of parameters, architecture, etc.) affects the performance of the proposed algorithm. In addition, the radar parameters and hardware specifications (i.e., number of chirps, memory capacity, etc.) influence the performance of the algorithm; these parameters were chosen according to [44].

As introduced in Section 2, the authors in [2] proposed an algorithm based on the wavelet entropy for shadow effect removal for human targets using UWB radars. This method proved to be effective in detecting two stationary human targets despite one person being in the shadowing region of the other. Hence, static filters (i.e., wavelet) were used. For each new possible deployment environment, an on-site adjustment is required: the number of filter levels and the wavelet function need to be tuned to accurately fit the application scenario. Therefore, the solution is not easily scalable because it needs expert intervention whenever a new context occurs. On the other hand, our proposal uses filters (i.e., weights of the convolutional layers) learned on a massive dataset (i.e., Imagenet dataset). This guarantees a high level of scalability and ease of deployment for different environments. In addition, it is not necessary to retrain the filters for new problems: only one dense layer needs to be fine-tuned for the incoming dataset, preserving the same structure of the pre-trained architecture, without requiring any expert intervention.

## 7. Conclusions and Future Works

In the case of multi-target detection using an FMCW radar, the target closest to the radar antennas partially reflects the energy of the electromagnetic wave, and the person farther from the radar antennas is not detected continuously, especially when in the shadowing region of the closest person. In this paper, a novel solution for the radar shadowing effect has been proposed. The solution is based on a CNN model that classifies the spectrogram images, obtained after a time-frequency analysis of the radar data, among one of two classes: One Target vs. Two Targets. The model is based on MobileNet and is loaded with the Imagenet weights. The best solution in terms of testing accuracy achieved 92.2% with a standard deviation of 2.86%, while the lightest (i.e., 1.06 million parameters) model attained 88.7% with a standard deviation of 2.39% over five splits of input data. The latter model uses 1.06 million parameters only and has a size of 5 MB. The inference time using a GPU is 1.64 ms. In future research, the authors plan to deploy the proposed solution on a Raspberry Pi and test the model in a real scenario. In addition, the distance between the visible target and the masked target should be assessed using a regression model. The proposed solution could be extended to different types of targets (e.g., cars, robots, pedestrians, etc.). This novel solution uses a supervised learning method; in other words, it already knows all the possible classes (One Target or Two Targets). If the situation of multiple shadowed targets needs to be addressed, it is theoretically possible by collecting enough data for every possible class. This method might not be practical because the number of classes could not be predicted beforehand. Therefore, the recommended procedure would be to shift the problem into an unsupervised problem. We are also considering an extension of this proposed approach; the goal is to detect and track two or more moving targets, with different inner distances, in a cluttered environment.

## Figures and Tables

**Table 1:** Position2Go radar specifications.

Parameters	Value
Sweep Bandwidth	200 MHz
Center Frequency	24 GHz
Up-Chirp Time	300 μs
Number of Samples/Chirp (Ns)	128
Number of Chirps/Frame (Nc)	32
Maximum Range	50 m
Maximum Velocity	5.4 km/h
Range Resolution	0.75 m
Velocity Resolution	0.4 km/h
Sampling Rate	42 KHz

**Table 2:** Sample of the available models.

Model	Num of Params (Million)	Top Accuracy (%)	Size (MB)	Inference Time (ms) on GPU
ResNet50	25.6	74.9	98	4.55
VGG19	143.6	71.3	549	4.38
MobileNet_V2	3.53	71.3	14	3.83
Small MobileNet_V3	2.0	73.8	12	3.57

**Table 3:** Data collection setup.

Class	Distance of Target A (d1) [m]	Distance of Target B (d2) [m]	Num of Meas. per Comb.
One Target	3	-	30
	5	-	30
	7	-	30
	9	-	30
	11	-	30
Two Targets	3	5	20
	5	7	20
	7	9	20
	9	11	20
	11	13	20

**Table 4:** Results over the four different models.

Model	Num of Params	Average Test	Inference Time	Size
	(Million)	Acc (%) ± STD	(ms) on GPU	(MB)
MobileNet_V2	2.3	81.5 ± 4.36	2.35	7.2
MobileNet_V3 Large	3.2	92.2 ± 2.86	2.23	18.2
MobileNet_V3 Small	1.6	90.9 ± 1.4	1.91	6.8
MobileNet_V3 Small Minimalistic	1.06	88.7 ± 2.39	1.64	5.0

## Data Availability

All data and code are available at: https://github.com/AmmarMohanna/ShadowingEffect (accessed on 23 December 2021).
